# Visualization of SNARE-Mediated Hemifusion between Giant Unilamellar Vesicles Arrested by Myricetin

**DOI:** 10.3389/fnmol.2017.00093

**Published:** 2017-03-31

**Authors:** Paul Heo, Joon-Bum Park, Yeon-Kyun Shin, Dae-Hyuk Kweon

**Affiliations:** ^1^Department of Genetic Engineering, College of Biotechnology and Bioengineering, Sungkyunkwan UniversitySuwon, South Korea; ^2^Department of Biochemistry, Biophysics and Molecular Biology, Iowa State UniversityAmes, IA, USA

**Keywords:** SNARE, membrane fusion, hemifusion, myricetin, calcium, neurotransmitter release

## Abstract

Neurotransmitters are released within a millisecond after Ca^2+^ arrives at an active zone. However, the vesicle fusion pathway underlying this synchronous release is yet to be understood. At the center of controversy is whether hemifusion, in which outer leaflets are merged while inner leaflets are still separated, is an on-pathway or off-pathway product of Ca^2+^-triggered exocytosis. Using the single vesicle fusion assay, we recently demonstrated that hemifusion is an on-pathway intermediate that immediately proceeds to full fusion upon Ca^2+^ triggering. It has been shown that the flavonoid myricetin arrests soluble N-ethylmaleimide-sensitive factor (NSF) attachment protein receptor (SNARE)-mediated vesicle fusion at hemifusion, but that the hemifused vesicles spontaneously convert to full fusion when the myricetin clamp is removed by the enzyme laccase. In the present study, we visualized SNARE-mediated hemifusion between two SNARE-reconstituted giant unilamellar vesicles (GUVs) arrested by myricetin. The large size of the GUVs enabled us to directly image the hemifusion between them. When two merging GUVs were labeled with different fluorescent dyes, GUV pairs showed asymmetric fluorescence intensities depending on the position on the GUV pair consistent with what is expected for hemifusion. The flow of lipids from one vesicle to the other was revealed with fluorescence recovery after photobleaching (FRAP), indicating that the two membranes had hemifused. These results support the hypothesis that hemifusion may be the molecular status that primes Ca^2+^-triggered millisecond exocytosis. This study represents the first imaging of SNARE-driven hemifusion between GUVs.

## Introduction

Membrane fusion constitutes the final step in the secretion and cargo transfer pathways between cellular compartments (Südhof and Rizo, 2011; Scheller, 2013; Rothman, 2014), and it is also essential in many cellular processes, including autophagy (Wang et al., 2016). When two membranes are approaching each other for fusion, free energy is required to overcome electrostatic repulsive forces, steric hindrances and the hydration force between two membranes. The soluble N-ethylmaleimide-sensitive factor (NSF) attachment protein receptor (SNARE) proteins comprise the molecular fusion machine. SNARE proteins provide the free energy required for fusion during the formation of a parallel four-helix bundle called the SNARE complex (Poirier et al., 1998; Sutton et al., 1998). In neurons, there are three SNARE proteins: syntaxin 1a (Stx1) and synaptosome-associated protein 25 (SNAP-25) on the plasma membrane and vesicle-associated membrane protein 2 or Syb2 (VAMP2) on the vesicle membrane. These three SNARE proteins constitute the minimal machinery for fusion between the synaptic vesicle and the presynaptic plasma membrane (Weber et al., 1998). Overwhelming evidence favors the zippering hypothesis, in which SNARE complex formation starts from N-termini and zippers progressively towards the membranes (Melia et al., 2002; Matos et al., 2003; Gao et al., 2012; Lou and Shin, 2016).

Many membrane fusion processes proceed via several sequential intermediates (Kozlov and Markin, 1983; Chernomordik and Kozlov, 2003; Chernomordik et al., 2006; Jahn and Scheller, 2006). When two membranes approach each other, they become locally connected by forming a hemifusion stalk. Proximal leaflets of bilayers are fused, but distal leaflets are separated at this stage. Hemifusion is also shown to be an on-pathway intermediate in SNARE-mediated membrane fusion (Lu et al., 2005; Xu et al., 2005). Subsequently, the stalk expands radially into a hemifusion diaphragm with the distal leaflets remaining separated, though it is possible that hemifusion expansion results in a dead-end product in Ca^2+^-triggered exocytosis (Diao et al., 2012; Hernandez et al., 2012). Finally, a fusion pore is opened within the hemifusion diaphragm, directly from the hemifusion stalk or from a point of membrane contact. Although hemifusion is considered to be an essential fusion intermediate, its experimental verification and characterization in biological membranes has been very difficult, yielding contradictory results (Zampighi et al., 2006; Wong et al., 2007; Fernández-Busnadiego et al., 2010; Zhao et al., 2016). After identification of the SNARE complex assembly and membrane fusion intermediate, Jahn and Scheller (2006) proposed that straining of lipids at the edge of an extended docking zone initiates fusion (Hernandez et al., 2012). Another cryo-electron microscopy study showed that Ca^2+^-triggered immediate fusion starts from a point-contact between membranes and proceeds to full fusion without discernible hemifusion intermediates (Diao et al., 2012). In both studies, stable hemifusion diaphragms were kinetically trapped and represented an off-pathway product. However, a study using super-resolution stimulated emission depletion microscopy observed membrane hemifusion directly in live chromaffin cells in real time (Zhao et al., 2016). An Ω-shaped hemifusion structure was observed in the live cells, and it was found that even the ‘kiss-and-run’ model can be explained by the competition between transitions of hemifusion/hemi-fission to full fusion or to full fission. Recently, we also showed that a stable hemifusion state can proceed to complete fusion and form a fusion pore almost synchronously with Ca^2+^ triggering (Heo et al., 2016). When a small molecule flavonoid (myricetin) that halts SNARE zippering in the middle (Yang et al., 2010) was removed from the SNARE complex intermediate with the enzyme laccase, hemifusion proceeded to full fusion. The speed of Ca^2+^-triggered fusion was comparable to that in neurons, and the pattern of release was reminiscent of the synchronous and asynchronous release of neuroexocytosis depending on the stage of Ca^2+^ arrival in the reconstituted systems (Heo et al., 2016). These results provide clear evidence that the hemifusion state is the bona fide intermediate enabling millisecond exocytosis.

Hemifusion mediated by SNARE proteins and myricetin was analyzed again using dynamic light scattering (DLS) spectroscopy (Yang et al., 2015). We verified hemifusion between vesicles in the presence of myricetin by simulating vesicle hydrodynamic radius changes during fusion and by cleaving SNARE proteins with proteinase K. In the present study, we aimed to visualize hemifusion between individual giant unilamellar vesicles (GUVs). GUVs are excellent objects for fluorescence microscopy visualization and analysis because GUV dimensions are larger than light microscopy’s intrinsic resolution limit. The mean diameter of GUVs is tens of μm, for which mean and dispersion values are strictly dependent on the method of GUV preparation. GUV size can be made comparable to the plasma membrane of a variety of cells. Membrane fusion processes, including the existence of the protein-free hemifusion diaphragm as a fusion intermediate, can be visualized using GUV and fluorescence microscopy (Lei and MacDonald, 2003; Heuvingh et al., 2004; Nikolaus et al., 2010). GUVs were also elegantly used to visualize the molecular interplay between membranes, accessory proteins and SNARE proteins (Bacia et al., 2004; Tareste et al., 2008; Hui et al., 2009; Malsam et al., 2012). Here, the hemifusion induced by SNARE proteins and myricetin was evaluated with confocal microscopy to directly visualize SNARE-mediated hemifusion.

## Materials and Methods

### Purification of SNARE Proteins

Neuronal SNAREs from *Rattus norvegicus*: SNAP-25 (amino acids 1–206), Syb2 (amino acids 1–116) and Stx1 (amino acids 1–288) were expressed in *Escherichia coli* CodonPlus-RIL (DE3) and purified by a glutathione S-transferase (GST) tag system. In brief, cell pellets were resuspended in PBS (pH 7.4) supplemented with 2 mM 4-(2-aminoethyl)-benzenesulfonyl fluoride, 2 mM ethylenediaminetetraacetic acid (EDTA) and 2 mM dithiothreitol (DTT). After sonication, the supernatant was mixed with GST-agarose beads at 4°C for 3 h. Excess PBS was used for washing, and each protein of interest was eluted in thrombin cleavage buffer (TCB, 50 mM Tris-HCl and 150 mM NaCl, pH 8.0). For transmembrane proteins, 0.2% Triton X-100 and 0.05% Tween 20 were added to PBS for the lysis and washing steps, and subsequently 1% *n*-octyl-beta-D-glucopyranoside (OG) was added to TCB instead of Triton X-100 at the elution step. All purified proteins were analyzed by SDS-PAGE and the Bradford assay.

### Reconstitution into LUV

We used a conventional SNARE reconstitution method to make proteoliposomes. 1-Palmitoyl-2-oleoyl-sn-glycero-3-phosphocholine (POPC), 1,2-dioleoyl-sn-glycero-3-phospho-Lserine (DOPS), 1,2-dipalmitoyl-sn-glycero-3-phosphoethanolamine-N-(7-nitro-2-1,3-benzoxadiazol-4-yl) (NBD) and 1,2-dioleoyl-sn-glycero-3-phosphoethanolamine-N-(lissaminerhodamine B sulfonyl, Rhod) were purchased from Avanti Polar Lipids Inc. A lipid mixture composed of PC:PS (95:5) was dried with nitrogen gas and further dried under vacuum overnight. For fluorescent GUVs, fluorescent lipids were incorporated into the mixture at the expense of PC. Large unilamellar vesicles (LUV) were formed by extruding the hydrated lipid mixtures through polycarbonate filters with a 100-nm pore size. SNARE proteins were mixed with liposomes at the indicated lipid/SNARE ratio while keeping the 0.8% OG concentration. The proteoliposomes were diluted with dialysis buffer (25 mM HEPES, 150 mM NaCl, pH 7.4) and dialyzed against 2 L dialysis buffer overnight at 4°C. Residual OG in samples was eliminated by SM2 Bio-Beads at 4°C for 30 mins. To compare reconstitution efficiency on GUVs depending on the lipid-to-protein (LP) ratio, SNARE proteins labeled with Alexa 647 dye through an amine reaction were used.

### SNARE-Reconstituted GUV Formation

The GUVs with SNARE proteins were generated by the electroformation method based on previous reports with modifications (Bacia et al., 2004; Tareste et al., 2008; Hui et al., 2009; Malsam et al., 2012). The process began after preparation of SNARE-embedded LUVs. After obtaining LUV pellets by centrifugation at 100,000 g at 4°C for 2 h, each pellet was resuspended in 10 mL low salt buffer (5 mM HEPES, 5 mM NaCl, pH 7.4). A droplet of the solution was applied to an indium tin oxide (ITO)-coated glass slide. After drying the lipid droplet, two ITO slides were assembled to form a 5 × 5 × 2 mm chamber (Supplementary Figure S1). The dried films were rehydrated with 200 mM sucrose while applying a sinusoidal electric field at 0.01 V and 10 Hz for 10 mins. The electric field was increased gradually by 0.1 V per 5 min up to 1.2 V, while maintaining the 10 Hz frequency. Then, 1.2 V (at 10 Hz) was applied for 6 h. Finally, 3 V (at 10 Hz) was applied for 15 min to detach the GUVs from the ITO slide. The SNARE-containing GUV mixture was incubated in 200 mM glucose solution to remove aberrant lipid aggregates. The efficiency of SNARE-containing GUV formation was estimated using fluorescent dye-labeled SNARE proteins and fluorescent lipids. GUVs were used for assays within 1 day of their formation. Note that residual OG strongly inhibited the formation of a dried film on the ITO slides and subsequent GUV formation. GUVs with no membrane proteins were generated following the same protocol as above, except that 2 μl of the lipid mixture in chloroform was directly spread on the ITO slides.

### Confocal Setup, GUV Fusion Assay and FRAP Assay

For membrane fusion assays, 5 μl of each t- and v-GUV preparation containing the binary acceptor complex (Syx1 and SNAP-25) and Syb2, respectively, were mixed with 5 μl buffer (25 mM HEPES, 400 mM KCl, 1 mM DTT, pH 7.4) and incubated at 37°C for 40 min. The GUVs deposited on glass slides were observed through a confocal laser-scanning microscope (LSM 700, Zeiss, Germany) equipped with a C-Apochromat 63×/1.2 water immersion objective. After focusing on the focal plane on the bottle-neck of a hemifused GUV pair, the fluorescence intensity distribution was obtained from the points on the line which cross the center of bottle-neck and meets the vesicles at opposite positons. The fluorescence intensities were normalized by the maximum fluorescence intensity. The fluorescence recovery after photobleaching (FRAP) assay was performed following the manufacturer’s instructions. After bleaching the regions of interest (ROI) at 100% power, fluorescence recovery was observed at the same power as before bleaching (~10% of maximum). ROI was ~10 μm in diameter to select entire single GUV. Further image analysis was performed with ZEN 2010 LSM software and ImageJ software (National Institutes of Health). The fluorescence recovery curve was fitted to an exponential decay function *f*(*t*) = *A*(1-exp(−*t*/*τ*)), where, *A* is the fraction of mobile component, *t* is the time passed after photobleaching and *τ* is the time constant. The lateral diffusion coefficient of lipids was calculated with following equation *D = r*^2^/4*τ*, where, *D* is diffusion coefficient, *r* is the radius of the photobleached GUV, and *τ* is the characteristic diffusion time.

## Results

### Reconstitution of SNARE Proteins into GUVs

**Figure 1:**
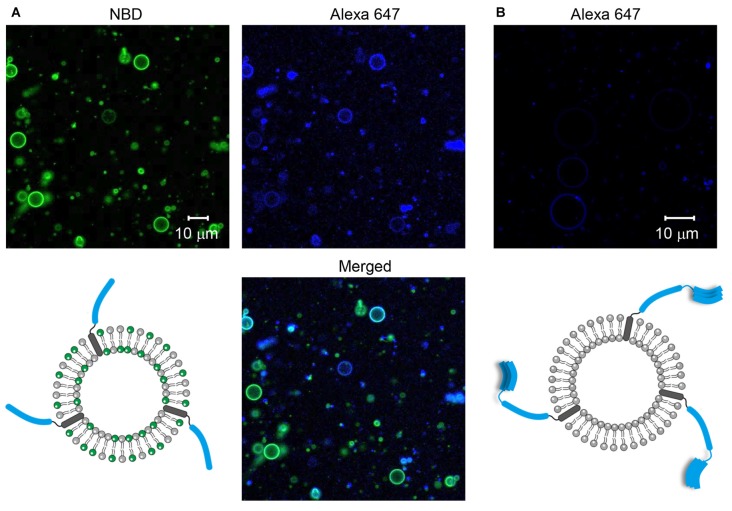
**Formation of soluble N-ethylmaleimide-sensitive factor (NSF) attachment protein receptor (SNARE)-containing giant unilamellar vesicles (GUVs). (A)** Syb2 labeled with Alexa 647 was reconstituted into GUVs containing nitro-2-1,3-benzoxadiazol-4-yl (NBD). **(B)** Syntaxin 1a (Stx1) labeled with Alexa 647 was reconstituted into GUVs that were not labeled with fluorescent dyes.

GUVs containing SNARE proteins were prepared through the electroformation method. To make sure that SNARE proteins were incorporated into GUVs, the SNARE proteins and/or GUVs were labeled with fluorescent dyes. First, Syb2 labeled with Alexa 647 was reconstituted into GUVs containing NBD. We observed many GUVs with the Alexa 647 fluorescent signal and the co-localized NBD signal (Figure 1A), indicating that Syb2-containing GUVs were well formed. Next, Stx1 labeled with Alexa 647 was reconstituted into the non-fluorescent GUVs to exclude the possibility that the fluorescence of circles derives from inadequate filtration of lipid fluorescence. We expected to observe the Alexa 647 signal on circles if the t-GUVs were formed as planned. Otherwise, no fluorescence or any signal from aggregates was expected. Though several amorphous dots representing protein aggregates were present, we found many circles representing GUVs containing Stx1 (Figure 1B).

We found that GUVs were not well formed when we tried to incorporate SNARE proteins above a certain limit. Because too few SNARE proteins on a GUV may not induce efficient GUV-GUV fusion, we tested several lipid-to-SNARE protein (LP) ratios to find an optimal condition for the efficient formation of SNARE-containing GUVs (Table 1). The efficiency of GUV formation was inversely proportional to the concentration of SNARE proteins. When the LP ratio was below 200 for Syb2 and below 500 for Stx1, we did not observe any GUV. In contrast, GUVs did not contain any SNARE proteins when the LP ratio was 4000, though many GUVs were observed. The 1000 LP ratio was optimal for both Stx1 and Syb2. For example, when Syb2 was incorporated into GUVs at an LP ratio of 1000, we observed tens of GUVs in a single focal plane, and approximately 73% of the GUVs contained fluorescently-labeled SNARE proteins (Table 1). Thus, we used an LP ratio of 1000 for further experiments.

**Table 1:** **The effect of lipid-to-protein (LP) ratio on the formation of giant unilamellar vesicles (GUVs) and reconstitution yields**.

SNARE proteins	LP ratio	GUV formation efficiency	SNARE-containing GUVs (%)
Syb2	200	−^1^	−
	500	+	98^2^
	1000	++	73
	2000	+++	44
	4000	+++	0
Stx1	200	−	−
	500	−	−
	1000	+	62
	2000	+++	41
	4000	+++	0

*^1^−, +, ++ and +++ represent the number of GUVs in a single focal plane. −, no GUV detected; +, a few GUVs; ++ 10–30 GUVs; +++ >50 GUVs. ^2^% of SNARE-containing GUVs = (number of GUVs with a signal for Alexa 647-labeled SNARE proteins/number of GUVs detected in the area) × 100*.

### GUV-GUV Fusion by SNARE Proteins

**Figure 2:**
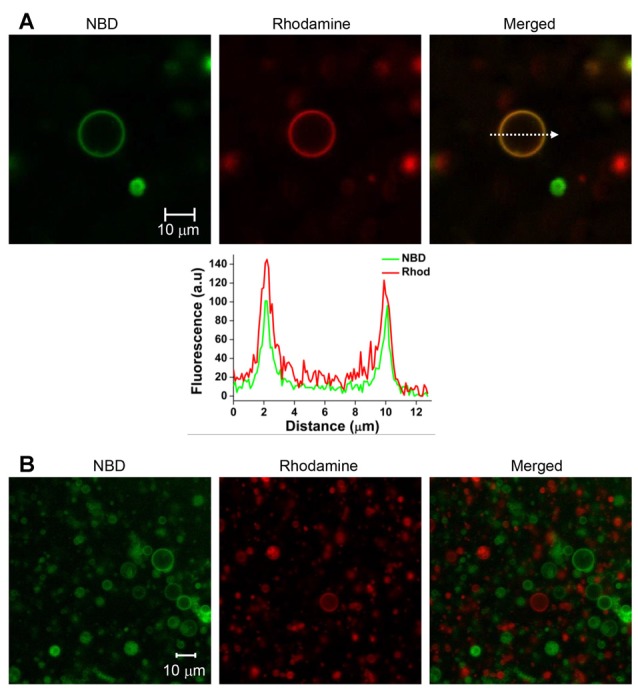
**SNARE-mediated full fusion between v- and t-GUVs. (A)** Representative images of fully fused GUVs. V-GUVs and t-GUVs were labeled with 3 mol % NBD and rhodamine, respectively. A fluorescence intensity profile (lower panel) was obtained, shown on the line indicated by an arrow in the merged GUV image. **(B)** Mixture of GUVs without SNARE proteins.

T-GUVs and v-GUVs containing the binary t-SNARE complex (Stx1 and SNAP-25) and Syb2, respectively, were separately prepared following the procedure described above at an LP ratio of 1000. T-GUVs and v-GUVs were labeled with rhodamine and NBD, respectively. After mixing equal amounts of t- and v-GUVs, the mixture was incubated at 37°C for 40 min. While major populations were unfused, we found that ~10% of the GUVs exhibited both NBD and rhodamine fluorescence, which is expected to happen when the two vesicles are fully fused (Figures 2A, Supplementary Figure S2). On the other hand, no vesicle exhibited both fluorescent signals when GUVs containing only fluorescent dyes (but no SNARE proteins) were mixed together (Figure 2B). This clearly indicated that GUVs did not spontaneously fuse in the absence of SNARE proteins. Thus, SNARE proteins reconstituted on GUVs mediated GUV-GUV fusion.

### Hemifusion between Two GUVs Arrested by Myricetin

**Figure 3:**
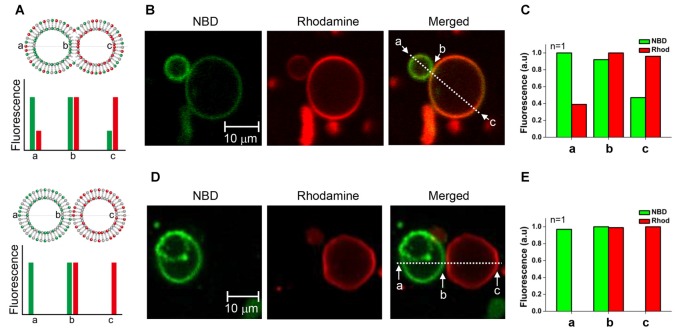
**Comparison of the hemifused vesicle pair and the docked vesicle pair. (A)** Schematic showing asymmetric fluorescence intensities depending on the location in the vesicle pair.** (B)** The fluorescently labeled v-GUVs (NBD, green) and t-GUVs (rhodamine, red) were mixed and incubated at 37°C for 40 min in the presence 1 μM myricetin. **(C)** The distribution of fluorescence intensity was measured at various regions of each GUV pair. **(D)** The fluorescently labeled v-GUVs and t-GUVs were mixed and incubated at 4°C for 60 min before taking the image. **(E)** The distribution of fluorescence intensity at various regions of the docked GUV pair.

We tested whether the membrane fusion intermediate arrested by myricetin was hemifused or not. It was expected that hemifused vesicles would show strong fluorescence intensities for both NBD and rhodamine near the stalk. Other regions of t-GUVs were expected to show a high rhodamine intensity with a lower NBD fluorescence intensity than that observed in the v-GUVs, and vice versa (Figure 3A, upper panel). If lipids were not mixed but the membranes were merely closely apposed, NBD fluorescence would not be detected in t-GUVs, and vice versa for v-GUVs (Figure 3A, lower panel).

The mixture of t- and v-GUVs was incubated at 37°C for 40 min in the presence of 1 μM myricetin. We observed hourglass-shaped vesicles forming in the presence of myricetin (Figures 3B, Supplementary Figure S3). One vesicle showed strong NBD fluorescence, while the other showed rhodamine fluorescence; this obviously represented the signal for the v- and t-GUVs, respectively. However, each vesicle also showed the fluorescence signal of the other vesicle, although the intensity was low. The intensity of each fluorescent signal was analyzed at three different positions: one near the stalk (designated b), one on the v-GUV (designated a) and one on the t-GUV (designated c). The asymmetry of the fluorescence intensities of NBD and rhodamine was dependent on the location of the GUV pair, and was consistent with what we expected for the hemifused GUVs (Figure 3C). On the other hand, GUV pairs were also observed when the mixture of t- and v-GUVs was incubated at 4°C (Figure 3D), which was a condition in which membrane fusion did not happen while docking of vesicles was allowed. Though the shape was similar to the hemifused vesicle pair, the fluorescent signals of NBD and rhodamine were not detected on opposite vesicles (Figure 3E). This result suggested that the hourglass-shaped GUV pairs enriched in the presence of myricetin were hemifused.

### Fluorescence Recovery after Photobleaching

Hemifusion between the vesicles in the GUV pair arrested by myricetin was confirmed with a FRAP assay. If two GUV outer leaflets are connected continuously, lipid molecules of the outer leaflets will diffuse laterally, leading to recovery of fluorescence after photobleaching. In contrast, diffusion of lipids from one GUV to another is not allowed if the GUV pair is simply docked, but bilayer leaflets are not connected between the two GUVs.

**Figure 4:**
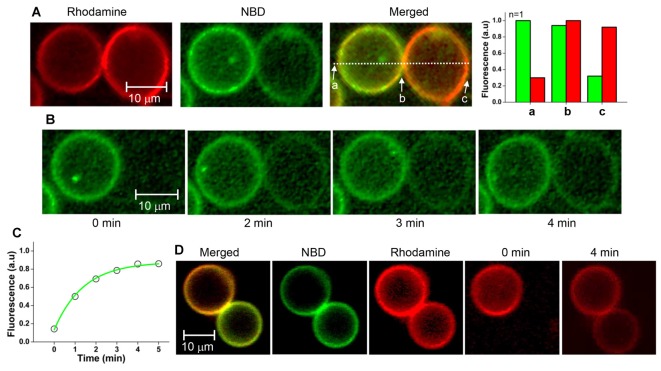
**Fluorescence recovery after photobleaching (FRAP) assay of the GUV pair arrested by myricetin. (A)** A GUV pair arrested by myricetin was selected, and the fluorescence intensity asymmetry dependent on the location was confirmed.** (B)** Representative images taken during FRAP experiments. The entire single v-GUV (the GUV on the right-hand side) was bleached, and its fluorescence recovery was monitored for 5 min.** (C)** Fluorescence recovery kinetics.** (D)** Representative FRAP images taken after t-GUV bleaching.

After hemifused GUV pairs were prepared in the presence of myricetin, GUV-GUV pairs that looked like hourglasses were selected. Hemifusion between a pair of GUVs was identified based on the fluorescence asymmetry as described above (Figure 4A). After photobleaching, the entire NBD fluorescence in the GUV at the right-hand side was measured as a function of time. The NBD fluorescence gradually recovered over time, suggesting that the NBD of the GUV on the left-hand side moved to the GUV on the right-hand side (Figure 4B). This result clearly indicated the GUV pair was hemifused. The lateral diffusion coefficient of the NBD between GUVs was calculated from the kinetics of fluorescence recovery (Figure 4C). It was determined to be 0.18 ± 0.03 μm^2^/s from 3 independent GUV pairs. The average decay constant *τ* was 71 s when the radius of a photobleached GUV was 7.15 μm. When the rhodamine fluorescence in one vesicle of the GUV pair was photobleached instead, fluorescence was recovered within a few minutes, consistent with NBD photobleaching (Figure 4D). These results suggested that the GUV pairs treated with myricetin were hemifused to allow lipid diffusion through the continuous outer leaflets of the two GUVs.

## Discussion

### SNARE-Driven GUV-GUV Fusion

GUVs are sufficiently large to be viewed using optical or fluorescence microscopy, and as such they are excellent samples to directly visualize and analyze the individual membrane fusion process. However, membrane protein incorporation, size control, and molecule encapsulation inside the GUVs are still challenging, although the electroformation-based method is relatively reproducible for protein-free GUV formation (Yamashita et al., 2002; Limozin et al., 2003; Chiantia et al., 2011; Dezi et al., 2013). It is likely that these difficulties have limited direct visualization of SNARE-driven fusion between GUVs, even in studies that have made use of GUVs for the analysis of membrane fusion (Bacia et al., 2004; Tareste et al., 2008; Hui et al., 2009; Nikolaus et al., 2010; Malsam et al., 2012). In the present study, all t- and v-SNARE proteins were successfully reconstituted in GUVs by optimizing the LP ratio and the GUV formation procedure. Reconstituted GUVs containing SNARE proteins enabled us to analyze membrane fusion intermediates occurring during GUV-GUV fusion with a fluorescence microscope.

The copy number of Syb2 in a synaptic vesicle with a 42-nm diameter is ~70, which corresponds to an LP ratio of ~176 in reconstituted proteoliposomes (Takamori et al., 2006; Ji et al., 2010). This LP ratio also corresponds to 5 × 10^6^ copies of Syb2 in a v-GUV with a diameter of 10 μm. However, we found that such a high protein density did not allow efficient GUV formation. Rather, higher LP ratios enabled more efficient GUV formation, though the probability that the GUVs contained SNARE proteins was lowered. GUV formation efficiency and SNARE incorporation appeared to be somewhat incompatible. We found that an LP ratio of 1000 and 2000 was optimal for both Syb2 and Stx1.

### Hemifusion Lipid Diffusion Coefficient

Our FRAP assay revealed a lipid diffusion coefficient of 0.18 μm^2^/s at 25°C. The diffusion constant of POPC (which was also used in our experiments) in multilamellar vesicles at 25°C is 7–10 μm^2^/s (Gaede and Gawrisch, 2003). This value is much smaller than the values for the cortical granule membrane and the plasma membrane (Wong et al., 2007). This small diffusion constant of lipids indicates the hemifused GUV pair does not share a wide area, and that only a small region is merged. The small shared area is most likely the bottleneck of the lateral lipid movement. It is not likely that the reconstituted SNARE proteins directly hindered the flow of lipid molecules because the protein density is too low to restrict lipid diffusion at such a low LP ratio as 1000. But, it is also possible that SNARE proteins that induced hemifusion do not dissipate from the stalk of hemifusion, restricting the lateral diffusion of lipids from one vesicle to the other (Chernomordik et al., 1998). Regardless of the exact reason for the small diffusion constant, it suggests that hemifusion arrested by myricetin does not expand to a wide area (i.e., the hemifusion diaphragm).

### Hemifusion in the Pathway to Fusion Pore Opening Observed by Utilizing Myricetin

We previously suggested that N-terminal half zippering might drive hemifusion. SNARE complex formation was arrested at the half-zippered state by a flavonoid (myricetin), and it was found that the membrane fusion intermediate arrested by myricetin corresponded to hemifusion in proteoliposome fusion. Though it is yet unclear how only half-zippering of the SNARE complex induced hemifusion, hemifusion arrested by myricetin could be converted to full fusion when the myricetin was removed from the SNARE complex by the enzyme laccase. The hemifusion was metastable, and Ca^2+^ could trigger immediate full fusion and content mixing. FRET-based bulk lipid-mixing assays (Yang et al., 2010), FRET-based single vesicle-vesicle lipid-mixing assays (Heo et al., 2016), FRET-based single vesicle-vesicle content-mixing assays (Heo et al., 2016), and DLS-based hydrodynamic radius change assays (Yang et al., 2015) were employed to investigate all of the features mentioned above. In the present study, it was shown that GUV-GUV fusion was also arrested in the hemifusion state by myricetin. This result suggested that hemifusion is a bona fide intermediate leading to fusion pore opening, and serves as the primed state for Ca^2+^-triggered millisecond exocytosis (Heo et al., 2016).

## Author Contributions

PH and D-HK devised the experiment. PH and J-BP performed the experiments. PH, Y-KS and D-HK wrote the article.

## Conflict of Interest Statement

The authors declare that the research was conducted in the absence of any commercial or financial relationships that could be construed as a potential conflict of interest.

## Supplementary Material

FIGURE S1 **Assembly of ITO slides**.

FIGURE S2 **Representative images of fully fused GUVs.** V-GUVs and t-GUVs were labeled with 3 mol % NBD and rhodamine, respectively. Fluorescence intensity profile (lower panel) was obtained on the line indicated by an arrow in the merged GUV image.

FIGURE S3 **Hemifused vesicle pairs exhibiting fluorescence intensity asymmetry. (A)** Images were taken in the same manner as in Figure 3, but in different experiments. The fluorescently labeled v-GUV (NBD, green) and t-GUV (rhodamine, red) were mixed and incubated at 37°C for 40 min in the presence 1 μM myricetin. The distribution of fluorescent intensity was measured at various regions of GUV pair. **(B)** Number of GUVs observed in this study. Major population (80%–90%) of GUVs in a focal plane is unfused free t- or v-GUVs even after optimization of GUV reconstitution scheme because of low probability of GUV-GUV fusion. Tens of glass slides from several independent experiments were used to obtain significant numbers of GUVs. We observed 161 and 117 GUVs in the absence and presence of 1 μM myricetin, respectively. Circles exhibiting both NBD and rhodamine fluorescence were counted as fully fused vesicles. Hourglass-shaped GUV pairs were counted by eye. Hemifusion was confirmed for 7 GUV pairs out of 79 hourglass-shaped GUV pairs by analyzing fluorescence intensity asymmetry. **(C)** Averaged fluorescence intensities which are asymmetrical depending on the locations on the GUV pair.
