# Supplementary figures and images for: Visualization of SNARE-Mediated Hemifusion between Giant Unilamellar Vesicles Arrested by Myricetin
Source: Front Mol Neurosci. 2017 Mar 31;10:93. doi: 10.3389/fnmol.2017.00093 (PMC5374201; doi:10.3389/fnmol.2017.00093)

**Fig. S1**

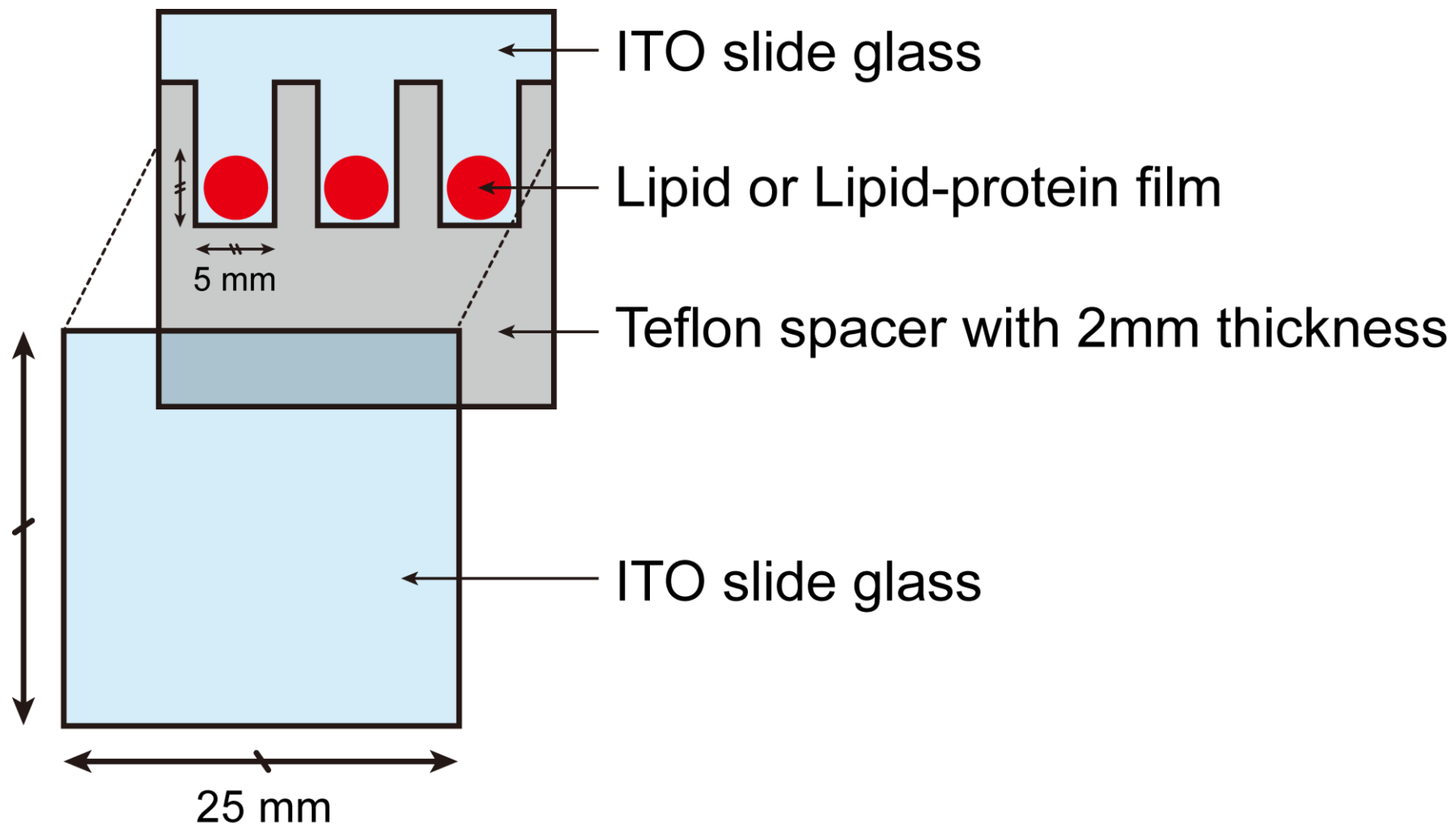

**Fig. S2**

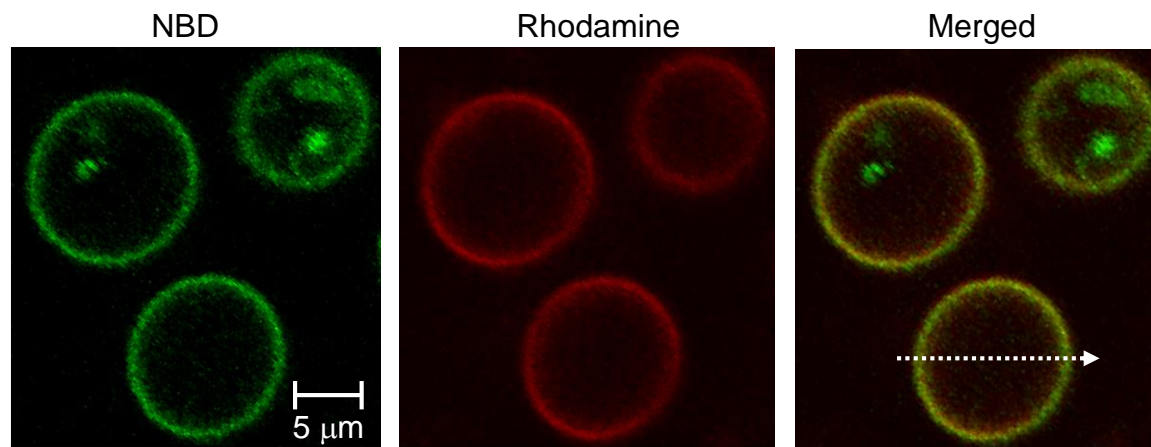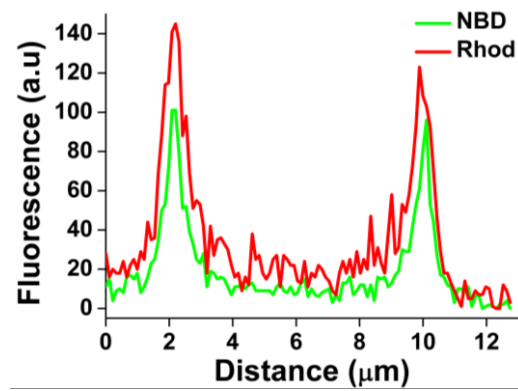

Fig. S3

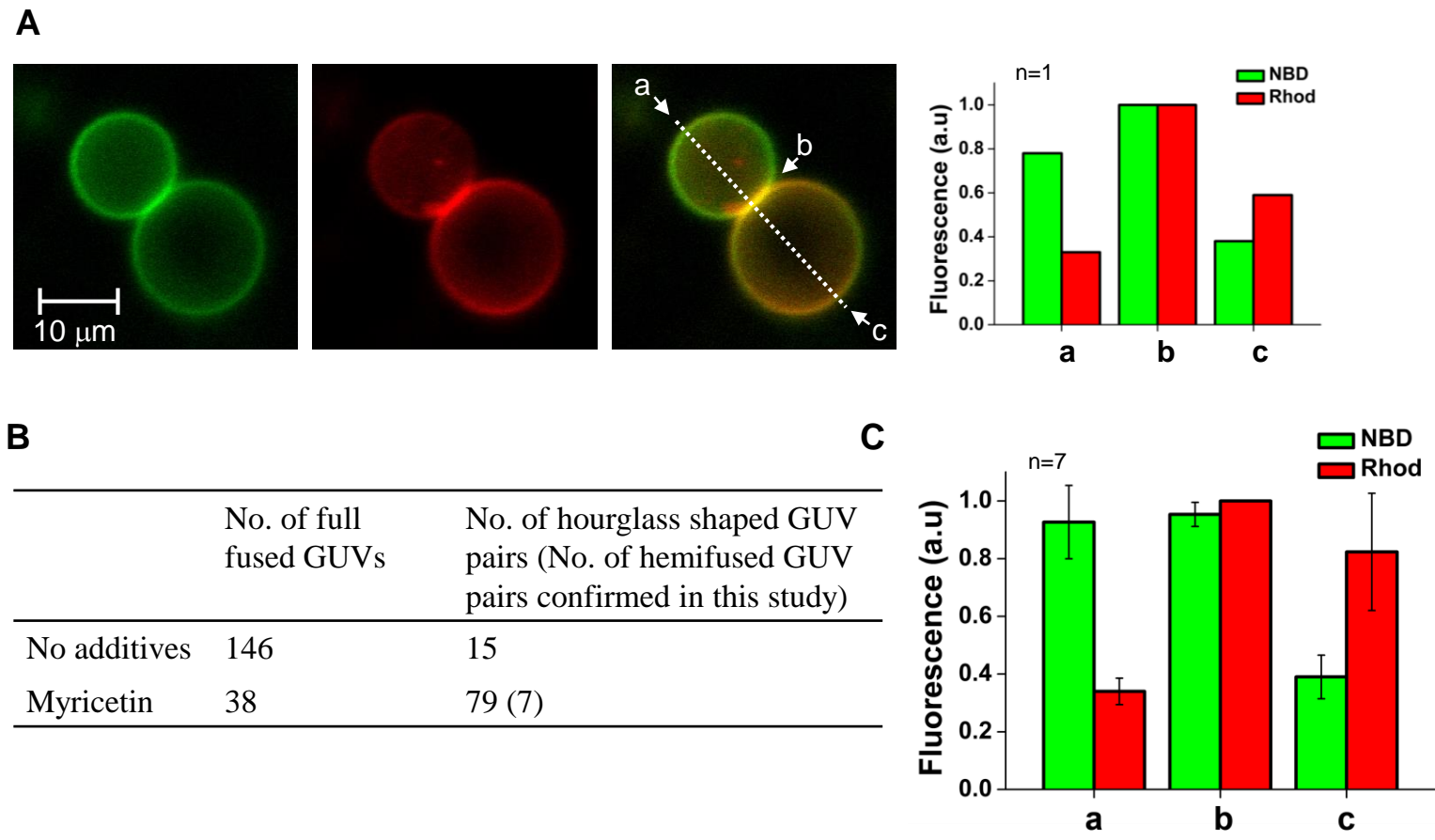

Supplement: FIGURE S1 — Assembly of ITO slides. [file Image_1.pdf]
